# Analysis of self-esteem and body image in affective psychotic disorder with onset in adolescence

**DOI:** 10.1192/j.eurpsy.2023.1221

**Published:** 2023-07-19

**Authors:** S. Susana Perez, I. Martin-Herrero

**Affiliations:** Psychiatry, Los Arcos del Mar Menor Universitary Hospital, murcia, Spain

## Abstract

**Introduction:**

In schizoaffective disorder, treatment with atypical antipsychotics is a first-line treatment option associated with treatment with mood stabilizers. One of the associated adverse effects is weight gain, which is sometimes associated with a deterioration of self-image and greater psychosocial impact.

**Objectives:**

1. To assess the personal perception and psychosocial adjustment in patients with affective psichotic disorder onset of symptoms in adolescence in current treatment with oral or IM aripiprazole. 2. To determine if there is variation in self-perception in patients with oral treatment compared to patients with long-term injectable treatment

**Methods:**

Patients with affective psichotic disorder onset in adolescence come to consultation. Retrospective data collection: 9 months. Cross-sectional assessment with E-PICA scale. SPSS21.0.

**Results:**

33 patients, 60% women (mean 38.6 years) and 40% men (40.46 years). Men consulted for psychiatric symptoms earlier and their diagnosis is earlier than in women (men: onset 16.8 years, ASD diagnosis 27.15; women: symptoms onset 20.5 years, diagnosis 37.1 years).

The use of ILD aripiprazole is observed in 54%, with a similar proportion in both sexes (men 53%, women 55%). In patients with oral aripiprazole, scores were obtained: mild psychosocial impact in 18%, all of them women; moderate impact 18% and severe impact in 9% of cases. While the results with ILD treatment: mild impact in 30% and moderate impact in 24% of cases, not observing severity scores in aripiprazole ILD.

**Conclusions:**

We observed a better body perception and self-esteem in women diagnosed with schizoaffective disorder and with long-term injectable aripiprazole treatment, assessing the psychosocial impact as mild. In men, the impact is greater, being observed more frequently in men with oral treatment. Continuity of follow-up and future studies will be necessary to determine other associated factors, as well as comparison with the use of other treatments.

**Disclosure of Interest:**

None Declared

